# Citrullination facilitates cross-reactivity of rheumatoid factor with non-IgG1 Fc epitopes in rheumatoid arthritis

**DOI:** 10.1038/s41598-019-48176-3

**Published:** 2019-08-19

**Authors:** Malgorzata Trela, Shantha Perera, Thomas Sheeran, Paul Rylance, Paul N. Nelson, Kesley Attridge

**Affiliations:** 1 Immunology Research Group, Research Institute in Healthcare Sciences, University of Wolverhampton, Wolverhampton, UK; 2 Department of Rheumatology, Royal Wolverhampton NHS Trust, Wolverhampton, UK; 3 Department of Nephrology, Royal Wolverhampton NHS Trust, Wolverhampton, UK; 4 School of Life & Health Sciences, Aston University, Birmingham, UK

**Keywords:** Rheumatoid arthritis, Autoimmunity

## Abstract

Rheumatoid factor (RF) and anti-citrullinated protein antibodies (ACPAs) are the two most prevalent autoantibodies in rheumatoid arthritis (RA), and are thought to have distinct autoantigen targets. Whilst RF targets the Fc region of antibodies, ACPAs target a far broader spectrum of citrullinated peptides. Here we demonstrate significant sequence and structural homology between proposed RF target epitopes in IgG1 Fc and the ACPA target fibrinogen. Two of the three homologous sequences were susceptible to citrullination, and this modification, which occurs extensively in RA, permitted significant cross-reactivity of RF+ patient sera with fibrinogen in both western blots and ELISAs. Crucially, this reactivity was specific to RF as it was absent in RF− patient and healthy control sera, and could be inhibited by pre-incubation with IgG1 Fc. These studies establish fibrinogen as a common target for both RF and ACPAs, and suggest a new mechanism in RF-mediated autoimmune diseases wherein RF may act as a precursor from which the ACPA response evolves.

## Introduction

Rheumatoid Arthritis (RA) is an autoimmune disease characterised by the production of diverse autoantibodies, of which rheumatoid factor (RF) represents a hallmark of the disease, being detectable in up to 80% of RA patients^1^. RF is known to target the Fc region of IgG1 antibodies, forming large immune complexes in synovial tissue^2^. These deposits initiate localised inflammatory processes and are propagated by the development of ectopic secondary lymphoid structures within the joint in which RF undergoes affinity maturation and isotype switching^3^.

The second major group of autoantibodies present in around 60–70% of RA patient sera constitute anti-citrullinated protein antibodies (ACPAs)^4^. These antibodies target epitopes that have been post-translationally modified by the deimination of arginine residues to citrulline. This modification is known to occur at a high frequency in RA synovium, as the peptidyl arginine deiminase (PAD) 2 and 4 isoforms responsible are overexpressed at this site. The resultant conformational changes produce neo-epitopes representing autoantigens to which tolerance has not been established^5,6^. Numerous ACPA targets have been identified to date, including fibrinogen, filaggrin, vimentin, type II collagen and α-enolase^7,8^. The recognition of multiple citrullinated autoantigens suggests that ACPAs play a crucial role in sustaining the autoimmune response in RA via epitope spreading and cross-reactivity^9,10^. Therapeutic targetting of the initiation of ACPA responses would therefore be highly desirable, as it could limit or help resolve RA responses, and could be utilised in other diseases in which ACPAs are known to play a role, such as psoriatic arthritis^11^ and pulmonary tuberculosis^12^. However, precisely how the autoantibody response to citrullinated antigens is initiated and propagated remains unclear.

In this study, we identify regions of sequence homology between the primary target of RF (IgG1 Fc), and the ACPA target, fibrinogen^13–17^, through *in silico* analysis. As arginine residues were present in the majority of these sequences, we utilized novel 3D modelling of citrullination to demonstrate significant sequence and structural homology between these regions. Finally, using sera from RA patients stratified based on ACPA and RF titres, we show that RF+ sera readily cross-reacts with fibrinogen after citrullination. These data suggest that cross-reactivity of RF with citrullinated auto-antigens represents a novel route for the initiation/propagation of ACPA responses in RA, a finding with potential relevance across a spectrum of autoimmune diseases in which RF is known to play a role, such as Sjögren’s syndrome and lupus.

## Results

### Sequence and structural homology between predicted RF epitopes in IgG1 Fc and the ACPA target fibrinogen

**Figure 1:**
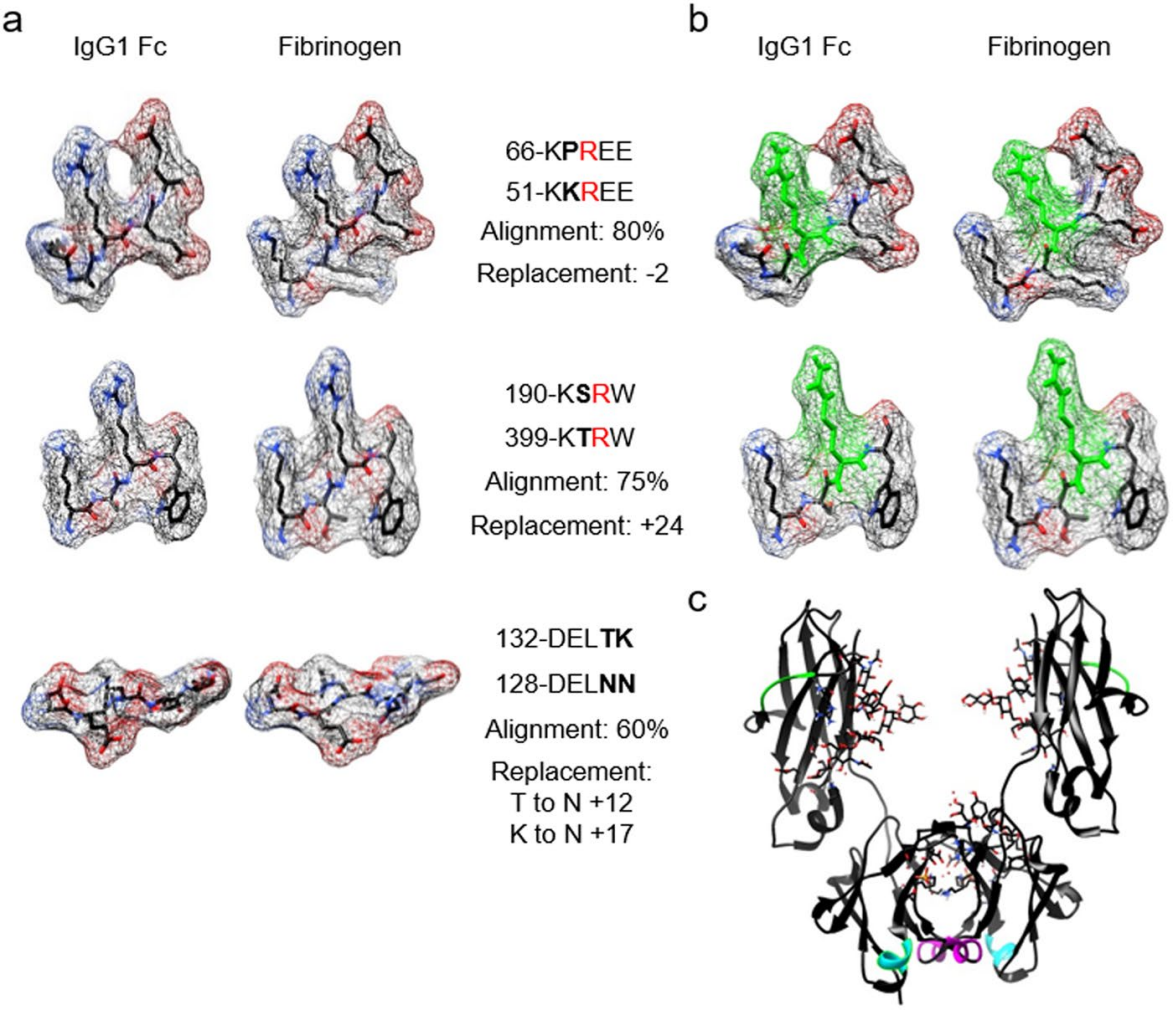
Regions of homology between predicted RF epitopes in IgG1 Fc and fibrinogen are targets for citrullination. (**a**) Three dimensional structures of regions of homology between IgG1 Fc and fibrinogen generated using PyMOL software. Sequences were scanned for regions of alignment using ExPASy SIM and LALIGN. Numerals indicate amino acid starting position and bold characters indicate amino acid substitutions. Characters highlighted in red identify arginine residues susceptible to citrullination. (**b**) Structures of regions of homology identified in (**a**) after modification of arginine residues to citrulline using PyTMs plugin. (**c**) Structure of the Fc region of IgG1 with the three predicted RF epitopes highlighted: KPREE (green), KSRW (cyan), and DELTK (magenta).

To determine whether cross reactivity of RF might play an important role in rheumatoid pathology, we searched for regions of homology between the sequences of IgG1 Fc (accession no. AF150959.1) and those of the fibrinogen β and γ chains (accession nos. P02675 and P02679, respectively) using ExPASy SIM and LALIGN software. These searches identified 3 regions of significant sequence homology, with molecular modelling using PyMOL further demonstrating significant conformational homology (Fig. 1a). Interestingly, we have previously identified 1 of these regions in IgG1 Fc (KPREE) to be a potential major RF-reactive site^18^. Of these 3 regions, 2 contained aligned arginine residues in the sequences of both IgG1 Fc and fibrinogen, identifying them as targets for citrullination. Modelling of citrullination of these sequences using the PyTMs plugin did not result in the loss of conformational homology (Fig. 1b). Further modelling of the full IgG1 Fc sequence determined that all 3 of the identified regions would be accessible to RF antibodies (Fig. 1c).

### Citrullination facilitates cross-reactivity of RF+ serum with fibrinogen in the absence of ACPAs

**Figure 2:**
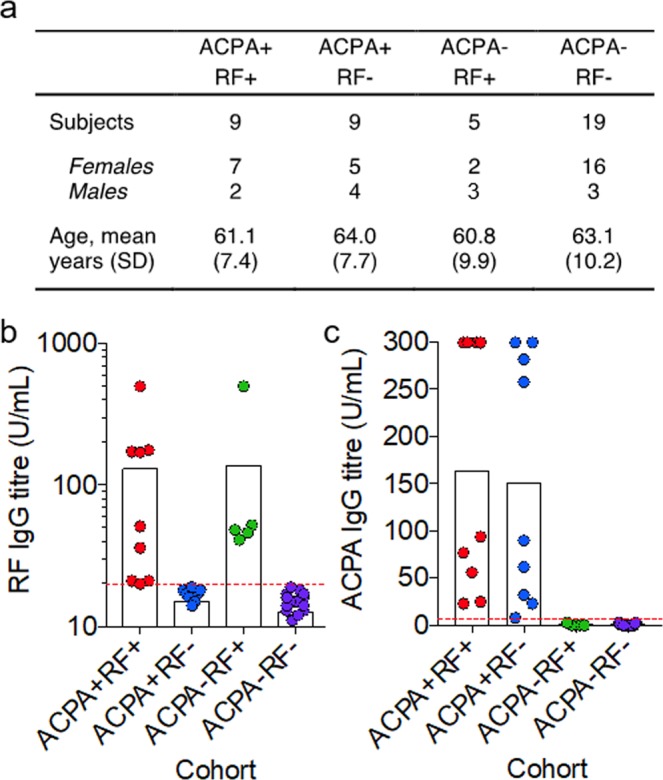
Stratification of rheumatoid arthritis patients according to serum titres of RF and ACPAs. (**a**) Patient characteristics amongst the four sera sub-types. (**b**) RF titres determined by IgG1 Fc-based ELISA for the four sera sub-types. Threshold for RF+ was set at 20 U/mL (red line). (**c**) ACPA titres determined by citrullinated protein-based ELISA for the four sera sub-types. Threshold for ACPA+ was set at 5 U/mL (red line).

**Figure 3:**
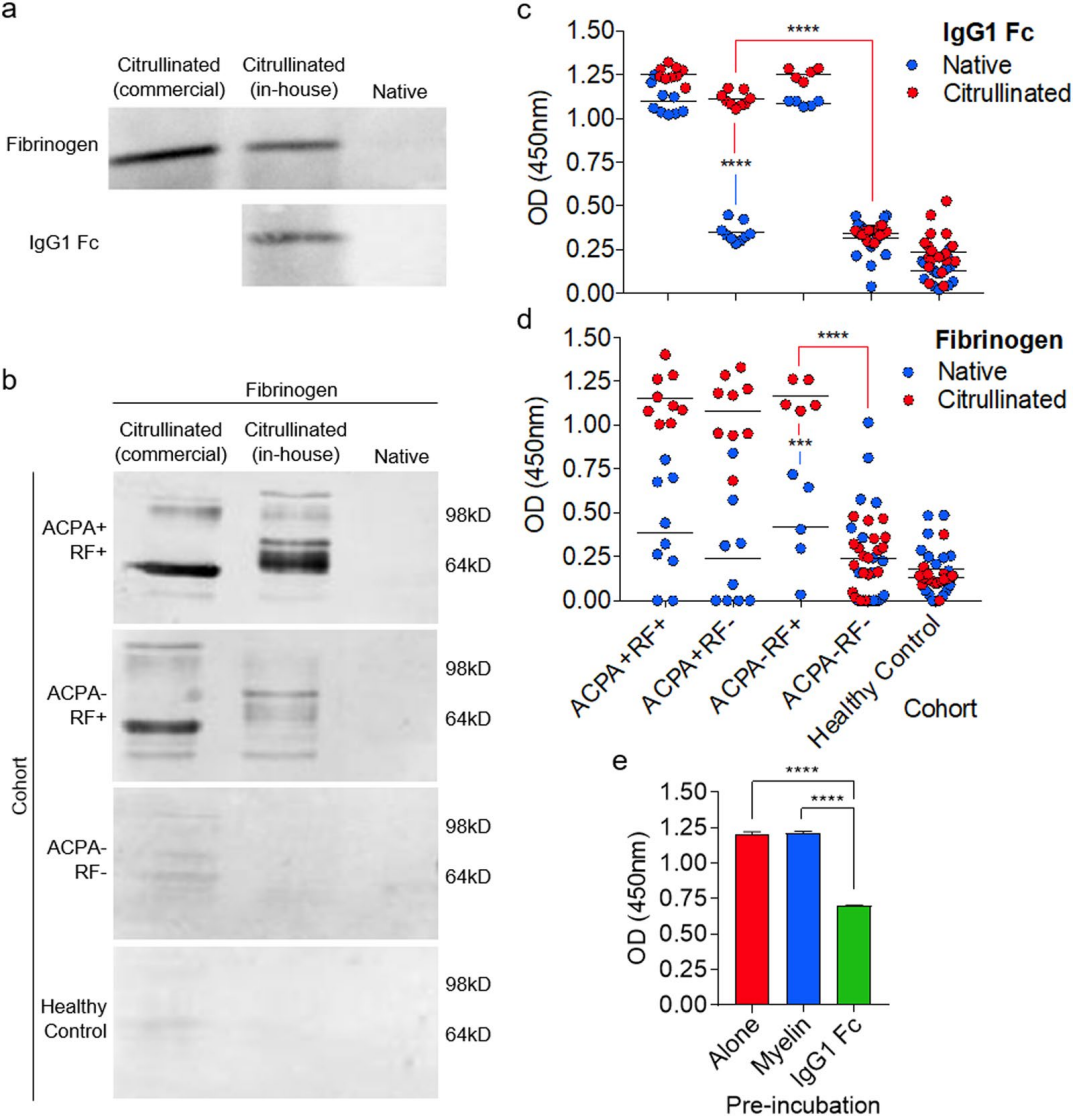
Citrullination facilitates cross-reactivity of RF+ serum with fibrinogen in the absence of ACPAs. (**a**) Native fibrinogen and IgG1 Fc were citrullinated in-house using a PAD enzyme cocktail or commercially (fibrinogen) and probed using a citrulline-specific reporter (rhodamine-phenylglyoxal). (**b**) Native or citrullinated fibrinogen was probed using sera from the indicated RA patient cohorts or healthy controls. Full length gels are shown in Fig. S2. (**c**) Graph shows serological response to native or citrullinated IgG1 Fc in the indicated RA patient cohorts or healthy controls, determined by ELISA. (**d**) Graph shows serological response to native or citrullinated fibrinogen in the indicated RA patient cohorts or healthy controls, determined by ELISA. (**e**) Graph shows reactivity of ACPA-RF+ sera with citrullinated fibrinogen after pre-incubation as indicated, determined by inhibition ELISA. Data in (**e**) represent three independent experiments. ****p < 0.0001; ***p < 0.001.

To determine whether homology between IgG1 Fc and fibrinogen in both their native and citrullinated forms would allow for cross reactivity of RF, we aimed to isolate RF+ sera from RA patients that had no detectable ACPAs, so that reactivity of sera samples with citrullinated fibrinogen could be specifically attributed to RF. We therefore recruited 42 RA patients (Fig. 2a) and determined their RF and ACPA titres by ELISA (Fig. 2b,c). Based on these data, sera were stratified into samples containing ACPAs and RF (ACPA + RF+), ACPAs alone (ACPA + RF−), RF alone (ACPA − RF+), or neither antibody (ACPA − RF−). Fibrinogen was then citrullinated using a PAD enzyme cocktail (confirmed using a citrulline-specific chemical probe in Fig. 3a), and assessed for reactivity with these sera subtypes by western blot. As expected, sera containing ACPAs reacted strongly with citrullinated fibrinogen (Fig. 3b, top panel). Despite the significant sequence and structural homology between IgG1 Fc and fibrinogen observed in Fig. 1, no detectable reactivity was observed for sera containing RF alone with native fibrinogen (Fig. 3b, second panel). However, when fibrinogen was citrullinated we observed binding of RF+ sera, even in the absence of detectable ACPAs, suggesting that citrullination facilitates cross-reactivity of RF with fibrinogen (Fig. 3b, second panel). This effect was RF-dependent, as reactivity with citrullinated fibrinogen was abolished in RF− sera from ACPA− RA patients (Fig. 3b, third panel) or healthy controls (Fig. 3b, bottom panel).

We next sought to confirm these data using ELISAs coated with fibrinogen or IgG1 Fc in their native or citrullinated forms. As expected, sera containing RF alone was able to react with native IgG1 Fc, whilst sera containing ACPAs alone only reacted with citrullinated IgG1 Fc (Fig. 3c). Accordingly, no reactivity with citrullinated IgG1 Fc was observed in the absence of ACPAs in RF− sera (Fig. 3c). In agreement with our western blot data, we observed limited reactivity of sera containing RF alone with native fibrinogen compared with RF− sera, whilst citrullination facilitated a significant increase in RF+ sera reactivity in the absence of ACPAs (Fig. 3d). Consistent with this effect being RF-dependent, reactivity with citrullinated fibrinogen was again abolished in RF− sera from ACPA− RA patients or healthy controls (Fig. 3d). Moreover, pre-incubation of ACPA-RF+ sera with the RF target IgG1 Fc significantly inhibited reactivity with citrullinated fibrinogen (Fig. 3e). No IgG was detectable in our citrullinated fibrinogen samples, excluding the possibility that contamination was responsible for the reactivity observed (Fig. S1). Collectively, these data provide direct evidence that RF is able to cross-react with the ACPA target fibrinogen, and that this reactivity is critically dependent on citrullination.

### RF reacts with citrullinated fibrinogen in the presence of ACPAs

**Figure 4:**
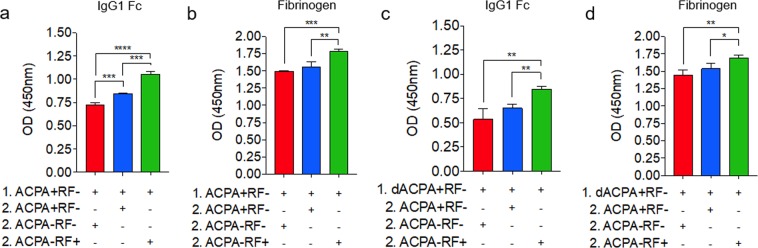
RF reacts with citrullinated fibrinogen in the presence of ACPAs. Graphs show serological response to citrullinated IgG1 Fc (**a**) or fibrinogen (**b**) when incubated with ACPA + sera (step 1), followed by incubation with the indicated RA patient sera-subtypes (step 2), determined by sequential ELISA. The same experiments were repeated using ACPA + sera digested with pepsin in step 1 (dACPA + RF−) for citrullinated IgG1 Fc (**c**) or fibrinogen (**d**). Data represent three independent experiments. ****p < 0.0001; ***p < 0.001; **p < 0.01; *p < 0.05.

In Fig. 1a, we identified the homologous sequences 132-DELTK and 128-DELNN in the IgG1 Fc and fibrinogen peptides, respectively. Because these sequences do not contain arginine residues, they are not susceptible to citrullination, and are not targeted by ACPAs. This suggests that when the full-length peptides are citrullinated, there remain free epitopes even in the presence of ACPAs to which RF may be reactive. To assess this, we established sequential ELISAs coated with citrullinated IgG1 Fc or fibrinogen that were first incubated with sera containing ACPAs alone, and then incubated with sera containing either antibody. For both citrullinated IgG1 Fc and fibrinogen, sequential incubation with ACPA + sera followed by RF+ sera lead to significantly higher reactivity than was observed with RF− sera (Fig. 4a,b). However, sequential incubation with primary ACPAs and secondary RF in this manner could result in the binding of RF to the Fc region of ACPA antibodies, which could be responsible for the increase in reactivity observed. To address this possibility, we repeated these experiments using ACPA + sera in the primary step that had been digested with pepsin to cleave Fc regions and affinity purified to isolate ACPA F(ab’)_2_ fragments (purity confirmed in Fig. S3). In these experiments, sequential incubation with ACPA F(ab’)_2_ + sera and sera containing ACPAs lead to no significant increase in reactivity, suggesting that all free ACPA target epitopes were saturated in the primary step (Fig. 4c,d). However, when sera containing RF were used in the secondary step there remained a significant increase in reactivity for both IgG1 Fc and fibrinogen (Fig. 4c,d). For fibrinogen, these data suggest that RF is able to interact with citrullinated fibrinogen even when in competition with ACPAs, by targeting additional non-ACPA target epitopes.

## Discussion

In this study, we utilised RF+ sera from RA patients seronegative for ACPAs to assess the ability of RF to react with citrullinated ACPA target proteins. Although these sera are composed of a heterogeneous mix of autoantibodies in addition to those assessed here, binding of ACPA- sera to citrullinated fibrinogen was only observed in RF+ samples, and this binding could be inhibited using the RF target IgG1 Fc, suggesting RF is the major determinant of reactivity. Previous studies have shown that only 4–8% of ACPA- RA sera react with the fibrinogen β chain when a single arginine residue is citrullinated^4^. Our data suggest that citrullination at multiple sites is therefore necessary to elicit reactivity with RF. It has been reported that citrullination can improve protein immunogenicity as a result of steric shifts and effects on relaxation dynamics in citrullinated proteins^6^. This finding is also in agreement with observations made by Hill and colleagues^19^, which suggest that the RA-associated HLA DRB1*0401 has a higher affinity for citrulline-modified peptides than for their native counterparts. These observations, alongside the fact that citrullination is a common feature of the rheumatoid joint, may explain why we observed more limited reactivity of RF+ RA sera with native fibrinogen, despite its homology with IgG1 Fc.

In recent years, a model for the initiation of rheumatoid pathology has been proposed that suggests ACPAs target citrullinated autoantigens, forming immune complexes containing Fc regions that initiate the production of RF^20^. According to this model, RF is dependent on ACPAs and should appear after the ACPA response is initiated. However, this model does not account for the production of RF in ACPA- patients. Broadening the model to include immune complexes containing non-citrullinated antigens reverses this order, with RF generated in response to immune complexes resulting in inflammation, influx of PAD enzymes, hypercitrullination and onset of the ACPA response^20^. In this model, ACPAs are therefore generated as an indirect result of the RF response. A number of studies have assessed the production of autoantibodies in the years before symptomatic RA, and it is clear that both RF and ACPAs can be produced during this period^21–26^. However, there is no clear consensus as to whether either antibody precedes the other, suggesting that both of the above models likely occur to some extent. In this study, we have shown that citrullination results in significant cross-reactivity of RF with a classical ACPA target, fibrinogen. Crucially, this observation suggests the possibility that RF could act directly as an ACPA precursor, blurring the line between what have always been considered distinct antibody classes in RA pathology. Therapeutic targeting of RF may therefore represent a route to limiting the ACPA response, which could help resolve inflammatory disease in RA and other autoimmune conditions such as lupus and Sjögren’s syndrome.

## Methods

### Patients with rheumatoid arthritis

Peripheral venous blood was collected from 42 Patients with RA recruited at Cannock Chase and New Cross NHS Hospitals, United Kingdom. All patients fulfilled the American College of Rheumatology criteria for the diagnosis of RA. Control sera (n = 20) were obtained from healthy individuals. Informed written consent was obtained from all participants and ethical approval was granted by the National Research Ethics Committee. All experiments were carried out in accordance with institutional guidelines.

### Measurement of RF and ACPA titres

Serum autoantibody titres were determined using commercial ELISA kits to assay RF (Abnova) and ACPAs (EuroDiagnostica) according to the manufacturers’ instructions.

### *In silico* analyses

The sequences of human fibrinogen (accession no. β: P02675, γ: P02679) and IgG1 Fc (accession no. AF150959.1) were obtained from the NCBI/GenBank database (www.ncbi.nlm.nih.gov/gquery/). Sequences were compared by pairwise BLAST analysis (www.ncbi.nlm.nih.gov/BLAST/) and scanned for regions of homology using ExPASy SIM (www.web.expasy.org/sim/) and LALIGN (www.ebi.ac.uk/Tools/psa/lalign/). Replacement scores were obtained using a substitution matrix developed by Tűdős *et al*.^27^.

Molecular models of homologous regions were created using PyMOL (www.pymol.org/pymol), with citrullination modelling achieved using the PyTMs plugin^28^. A model of the full human IgG1 Fc (entry: 5JII) was obtained from the Protein Data Bank (www.rcsb.org), processed with PyTMs plugin, and enhanced using UCSF Chimera (www.cgl.ucsf.edu/chimera).

### *In vitro* citrullination

Native human fibrinogen (Bio-Rad) and IgG1 Fc (Abcam) were citrullinated by 3 h incubation at 55 °C with active PAD enzyme cocktail (0.5 µg/µL; P312-37C-25; SignalChem). Native control samples were prepared using the same protocol, in the absence of enzymes. Commercially citrullinated human fibrinogen (CAY400076; Cambridge Bioscience) was used as a positive control. Citrullination was confirmed by labelling with rhodamine-phenylglyoxal (Cayman Chemical) according to the protocol described in Bicker *et al*.^29^, and imaging.

### Gel electrophoresis and Western blot

Protein samples in loading buffer were heated at 96 °C for 30 min, separated on a 6% SDS-PAGE, and transferred onto nitrocellulose by electroblotting. Membranes were blocked with 5% milk powder for 1 hr before probing with patient serum samples (1/200) overnight at 4 °C. Bands were visualized by incubation with HRP-labelled goat F(ab’)2 anti-human IgG conjugate (STAR97P; Serotec) for 1 hr, followed by enhanced chemiluminescence detection system (Amersham), and imaging.

### ELISA

Screening of fibrinogen and IgG1 Fc was conducted as previously described^30^. Briefly, proteins were coated (8 µg/mL) onto 96-well multiscreen plates (2 HB Immulon; Dynex) and incubated overnight at 4 °C. Plates were blocked with 2% BSA for 1 h at 37 °C. Sera were then used alone or sequentially (1/200; 1 hr), as indicated, followed by incubation with HRP-labelled F(ab’)2 anti-human IgG conjugate (6 µg/mL; 1 hr), and TMB (Sigma Aldrich) before reading at 450 nm. For inhibition assays sera were pre-incubated alone, with myelin (Sigma Aldrich; 10 µg/mL) or IgG1 Fc (10 µg/mL) for 1 hr at 37 °C.

### Antibody digestion with pepsin

ACPA + RF− sera were incubated with active pepsin (Sigma Aldrich) at a 20:1 ratio (serum:enzyme) for 1 hr at 37 °C. F(ab’)_2_ fragments were negatively selected using Protein G affinity columns (Thermo Fisher) and filtered over a 30 kDa membrane (Millipore) to remove low molecular weight contaminants.

### Statistical analyses

Results are presented as mean and SEM. Unpaired *t* tests with a 95% confidence interval were used for pairwise comparisons to determine statistical significance.

## Supplementary information

Trela et al Supplementary Information

## Data Availability

The datasets generated during the current study are available from the corresponding author on reasonable request.
