# Supplementary material for: Citrullination facilitates cross-reactivity of rheumatoid factor with non-IgG1 Fc epitopes in rheumatoid arthritis
Source: Sci Rep. 2019 Aug 19;9:12068. doi: 10.1038/s41598-019-48176-3 (PMC6700074; doi:10.1038/s41598-019-48176-3)
Supplement: Supplementary file 1 — Trela et al Supplementary Information [file 41598_2019_48176_MOESM1_ESM.pdf]

Supplementary Information

Citrullination facilitates cross-reactivity of rheumatoid factor with non-IgG1 Fc epitopes in rheumatoid arthritis

Malgorzata Trela[1], Shantha Perera[1], Thomas Sheeran[2], Paul Rylance[3], Paul N. Nelson[1†] and Kesley Attridge[1,4,*]

1. Immunology Research Group, Research Institute in Healthcare Sciences, University of Wolverhampton, Wolverhampton, UK
2. Department of Rheumatology, Royal Wolverhampton NHS Trust, Wolverhampton, UK
3. Department of Nephrology, Royal Wolverhampton NHS Trust, Wolverhampton, UK
4. School of Life & Health Sciences, Aston University, Birmingham, UK

†Deceased 30th June 2016
*Corresponding author:

Kesley Attridge PhD
MA Building Room 209
University of Wolverhampton
Wulfruna Street
Wolverhampton
WV1 1LY
Tel: +44 1902 32 1149
Email: k.attridge2@wlv.ac.uk

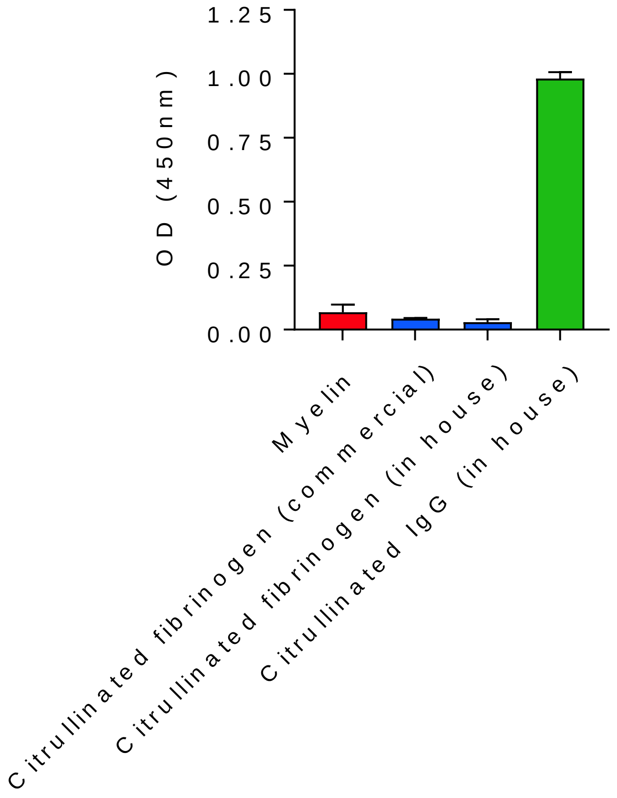

Supplementary Figure S1. IgG is not detectable in fibrinogen samples used in this study. Native fibrinogen and IgG were citrullinated in-house using a PAD enzyme cocktail or commercially (fibrinogen). Graph shows reactivity of a polyclonal anti-IgG antibody with myelin (negative control), citrullinated fibrinogen, and citrullinated IgG (positive control), determined by ELISA. Data represent three independent experiments.

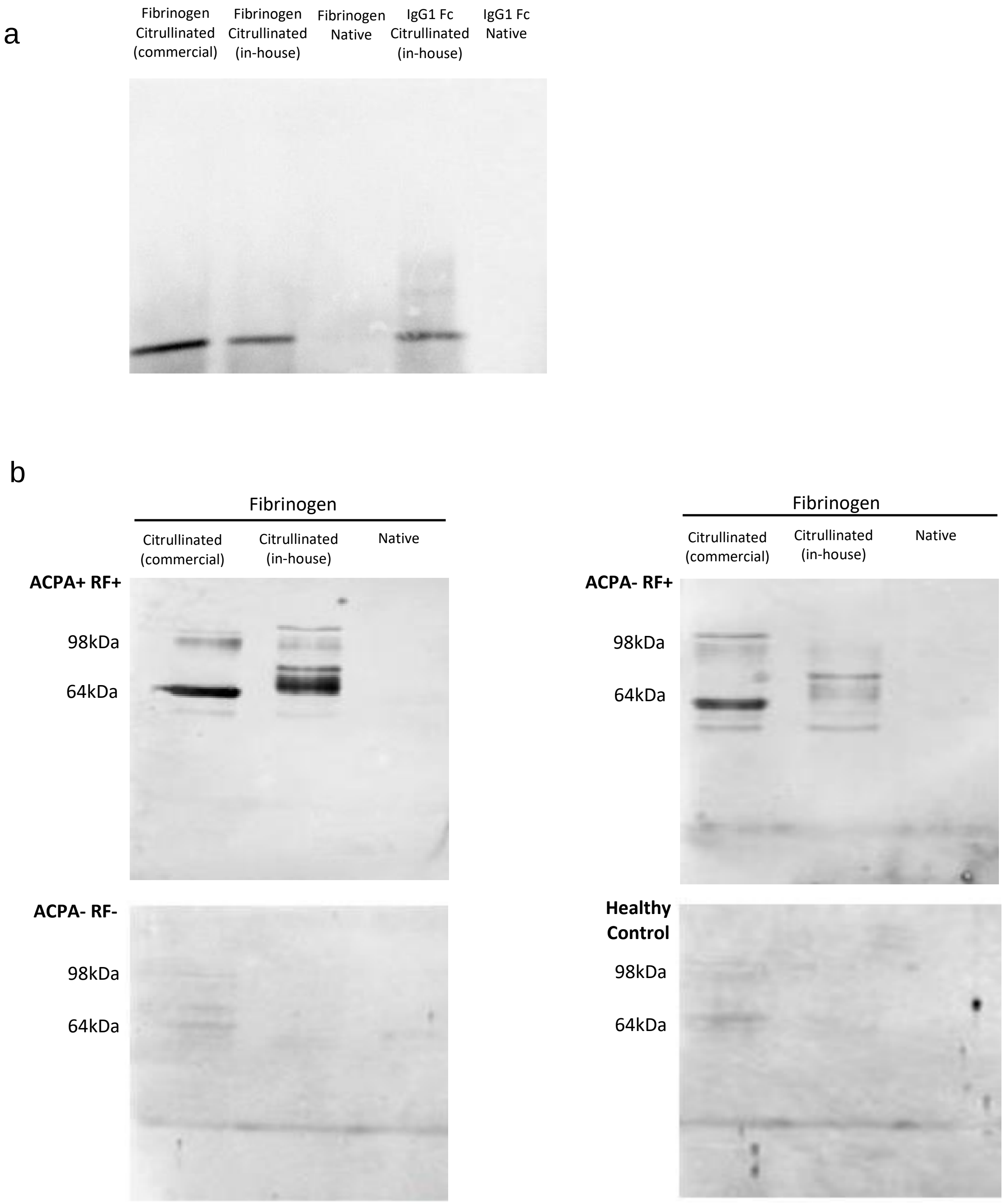

Supplementary Figure S2. Full length gels for western blot images shown in Figure 3. Each lane was loaded with a single purified protein sample, as indicated. (A) Identification of in-house citrullinated fibrinogen and IgG1 Fc with a rhodamine-phenylglyoxal citrulline-specific reporter probe. (B) Native and citrullinated fibrinogen was probed using sera from the indicated RA patient cohorts.

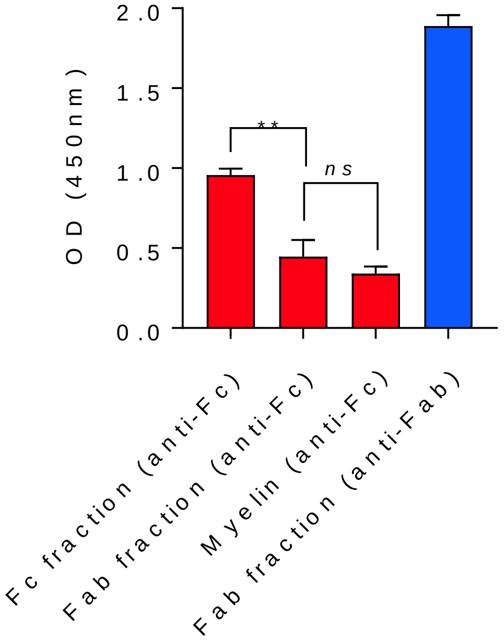

Supplementary Figure S3. Fc is not detectable in the purified $F(ab')_2$ fraction of pepsin-digested ACPA+RF- sera. Graph shows reactivity of an anti-IgG Fc antibody with the Fc and $F(ab')_2$ fractions isolated after pepsin digestion of ACPA+RF- sera, or with myelin as a negative control (red bars), determined by ELISA. The reactivity of an anti-IgG Fab antibody with the $F(ab')_2$ fraction is also shown (blue bar). Data represent three independent experiments. ** $p<0.01$; *ns* not significant.

Supplementary Methods

ELISA

Proteins were coated onto 96-well multiscreen plates (fibrinogen at 8 µg/mL; ACPA+RF- Fc and ACPA+RF- $F(ab')_2$ fractions at 1/200) and incubated overnight at 4°C. Plates were blocked with 2% BSA for 1h at 37°C. For detection of IgG contamination, plates were incubated with goat anti-human IgG Fc antibody for 1hr (Bethyl; 0.5 µg/mL), followed by incubation with HRP-labelled anti-goat IgG (0.05 µg/mL) for 45mins. For assessment of antibody digestion, plates were incubated with anti-human HRP conjugates against IgG Fc (Bethyl; 0.05 µg/mL) or IgG Fab (10 µg/mL) for 1hr. Plates were incubated with TMB (Sigma) and read at 450nm. Where indicated, myelin (Sigma Aldrich) was used as a negative control and IgG (Abcam) was used as a positive control.
